# One Stone, Two Birds: N6-Methyladenosine RNA Modification in Leukemia Stem Cells and the Tumor Immune Microenvironment in Acute Myeloid Leukemia

**DOI:** 10.3389/fimmu.2022.912526

**Published:** 2022-06-02

**Authors:** Xianfeng Ouyang, Yuping Gong

**Affiliations:** ^1^ Department of Hematology, West China Hospital, Sichuan University, Chengdu, China; ^2^ Department of Hematology, Jiujiang First People’s Hospital, Jiujiang, China

**Keywords:** N6-methyladenosine, RNA methylation, acute myeloid leukemia, leukemia stem cells, tumor immune microenvironment

## Abstract

Acute myeloid leukemia is the most common acute leukemia in adults, with accumulation of abundant blasts and impairment of hematogenic function. Despite great advances in diagnosis and therapy, the overall survival of patients with acute myeloid leukemia remains poor. Leukemia stem cells are the root cause of relapse and chemoresistance in acute myeloid leukemia. The tumor immune microenvironment is another trigger to induce recurrence and drug resistance. Understanding the underlying factors influencing leukemia stem cells and the tumor immune microenvironment is an urgent and unmet need. Intriguingly, N6-methyladenosine, the most widespread internal mRNA modification in eukaryotes, is found to regulate both leukemia stem cells and the tumor immune microenvironment. Methyltransferases and demethylases cooperatively make N6-methyladenosine modification reversible and dynamic. Increasing evidence demonstrates that N6-methyladenosine modification extensively participates in tumorigenesis and progression in various cancers, including acute myeloid leukemia. In this review, we summarize the current progress in studies on the functions of N6-methyladenosine modification in acute myeloid leukemia, especially in leukemia stem cells and the tumor immune microenvironment. We generalize the landscape of N6-methyladenosine modification in self-renewal of leukemia stem cells and immune microenvironment regulation, as well as in the initiation, growth, proliferation, differentiation, and apoptosis of leukemia cells. In addition, we further explore the clinical application of N6-methyladenosine modification in diagnosis, prognostic stratification, and effect evaluation. Considering the roles of N6-methyladenosine modification in leukemia stem cells and the tumor immune microenvironment, we propose targeting N6-methyladenosine regulators as one stone to kill two birds for acute myeloid leukemia treatment.

## 1 Introduction

Acute myeloid leukemia (AML) is a hematologic malignancy characterized by abnormal proliferation of blasts or immature progenitor cells in the bone marrow, blood and other tissues with arrested differentiation and apoptosis disorder, generating suppression of normal hematopoiesis (1).

Over the last few decades, intensive induction chemotherapy, such as the combination of anthracycline and cytarabine, consisting of the “3+7” regimen, has been the standard scheme for fit patients (2). According to age, physical state, comorbidity, complications, and hematological and genetic prognostic indicators, current treatment approaches involve combination chemotherapy, hypomethylating agents (HMAs), BCL-2 inhibition, targeted therapy, immunotherapy, and/or hematopoietic stem cell transplantation (HSCT) (3, 4). In this model of treatment, complete remission rates can achieve 60-80% in younger patients and 40-60% in older patients (1, 5, 6).

The survival rates of patients with AML vary from person to person, which is attributed to differences in their ages and clinical and genomical prognostic characteristics. Nevertheless, most patients will eventually relapse (3). Leukemia stem cells (LSCs) are deemed to be resistant to chemotherapy, leading to disease recurrence (7). When transplanted into immunodeficient mice, LSCs have the ability to trigger the disease and can maintain self-renewal (8).

Immune evasion is another cause of the relapse of leukemia. It is widely known that the human immune system can identify and kill non-self components, such as leukemia cells (9). However, in fact, AML blasts, including LSCs, develop multiple mechanisms to escape host immune surveillance and eradication (10, 11). In addition to the feature of tumor initiation, immune evasion is an even more vital competence of cancer stem cells (CSCs) (12, 13). LSCs were first identified as CSCs employing combined immunodeficient mice (14). CSCs regulate the tumor immune microenvironment (TIME) *via* immune evasion, such as expressing immunosuppressive molecules and recruiting immunosuppressive cells (15). Likewise, the TIME can cooperate with CSCs to promote tumor progression (16–19).

Recently, research on the functions of RNA epigenetic modification in AML has grown vigorously. Above all, the N6-methyladenosine (m^6^A) RNA modification is of the greatest concern (20, 21). First, fat mass and obesity associated protein (FTO) was found to promote leukemia initiation and progression, and is considered to have carcinogenic activity. A variety of leukemia cells highly express FTO in several subtypes of AML (22). Subsequently, methyltransferase-like 3 (METTL3), methyltransferase-like 14 (METTL14), Wilm’s tumor 1-associated protein (WTAP), and AlkB homolog 5 (ALKBH5) were reported to be related to myeloid leukemia (23–25). Meanwhile, many studies have found that m^6^A participates in the regulation and maintenance of the stemness of CSCs (26–28). m^6^A modification is also pivotal for the self-renewal of LSCs (21). In addition, m^6^A modification also involves the modulation of the TIME in various cancers, as well as in AML (29–32).

In this review, we spark new ideas about m^6^A modification in LSCs and the TIME for better clinical treatment implications in AML.

## 2 LSCs and the TIME

Despite achieving complete remission after chemotherapy, AML patients ultimately die of recurrence due to chemotherapy-resistant LSCs (33–35). Lapidot et al. first identified AML-initiating cells as LSCs that had the ability to repopulate human leukemia in SCID mice. Analogous to normal hematopoietic stem cells (HSCs), CD34+CD38- is the typical phenotype of LSCs (14). In addition, LSCs can be present in CD34+CD38+ cellular compartments and even in the CD34- subpopulation (36, 37). In addition to the properties of self-renewal, proliferation and differentiation, LSCs are characterized by cell cycle quiescence, low energy requirements, hypomethylated state and chemoresistance (8, 38). Apart from CD34 and CD38, there are various cell-surface markers on LSCs. For instance, TIM-3, CLL-1, CD47, CD70/CD27, CD96, CD123, CD244, CD200 and CD93 (39–48).

T cell immunoglobulin and mucin protein 3 (TIM-3), which is expressed in AML cells, secretes its ligand Galectin-9 (Gal-9) to constitute a TIM-3/Gal-9 autocrine loop. The loop is capable of promoting the self-renewal of LSCs by activating both NF-kB and the Wnt/β-catenin signaling pathways (40).In addition to Gal-9, TIM-3 in AML cells produces soluble TIM-3 (sTIM-3), which can attenuate the release of IL-2. The cytokine IL-2, secreted by T cells, is pivotal to the activation of cytotoxic T cells and NK cells (49). Because of the expression of TIM-3 in various immune cells, such as T cells, NK cells, DCs and mast cells, Gal-9 secreted by LSCs can combine with TIM-3 in immune cells, giving rise to a suppressive tumor immune microenvironment. The accumulation of β-catenin in LSCs is essential for self-renewal and progression. Additionally, β-catenin even lures the expression of a variety of immune checkpoints, including TIM-3, to suppress the host immune system (50). Likewise, TIM-3 also activates the PI3K/mTOR signaling pathway, resulting in the accumulation of hypoxia-inducible factor 1-alpha (HIF1α), which can induce programmed cell death-ligand 1 (PD-L1) expression in tumor cells to escape adaptive immunity (51, 52).

CD47 is considered a marker of LSCs in AML. Elevated expression of CD47 on LSCs can restrain phagocytosis of macrophages, a kind of phagocyte expressing SIRPα. LSCs can initiate the signaling “Don’t eat me” through the CD47-SIRPα interaction to escape from host innate immune attack (42). CD200 is highly expressed in LSCs. Herbrich and his colleagues found that CD200+ AML cells could inhibit T cell cytokine secretion, change T cell composition and cell cycle, interrupt T cell metabolism, and weaken the macrophage response to AML cells. Furthermore, CD200 monoantibody therapy can counteract these effects, as foresaid (47). Poly-ADP-ribose polymerase 1(PARP1) in LSCs is able to suppress the expression of NKG2D ligand (NKG2DL), leading to evasion of NK cell killing (53).

Taken together, LSCs can take advantage of various mechanisms to escape from immunological surveillance in the tumor microenvironment (TME). In turn, immune cells in the TME can add further weight to promote and maintain the self-renewal and progression of LSCs. That CSCs can merely establish and self-renew in immunodeficient mice such as NOD/SCID mice is a most convincing example to elucidate that immune selection may play a crucial part in CSCs implantation and maintenance (15). Depreter and colleagues reported that immune dysregulation could be responsible for the initiation and maintenance of LSCs in pediatric AML (54). PD-1, CTLA-4, TIM-3 and LAG-3 overexpression in bone marrow T cells contributes to AML relapse after allogeneic HSCT. The function of these T cells in bone marrow is exhausted due to the declining ability to produce cytotoxic cytokines (55). Xu et al. found that TIGIT and PD-1 were simultaneously overexpressed on CD8+ T cells in the bone marrow of AML patients, giving rise to the immunosuppressive microenvironment (56). Additionally, cytokine signaling in the TIME also plays a role in the regulation of LSCs (57).

In brief, LSCs interact with the TIME in a variety of ways to enhance and maintain AML progression and chemoresistance, ultimately resulting in disease relapse.

## 3 m^6^A Modification in LSCs and the TIME

It is well known that epigenetic modification plays pivotal roles in the genesis and development of all kinds of cancers, including AML. DNA methylation, histone modification, and chromatin remodeling are common epigenetic modifications. Recently, RNA modification has increasingly served as a hotspot of epigenetic studies. In particular, N6-methyladenosine (m^6^A) RNA modification, referring to methylation on the sixth atom of adenosine, is the most intriguing and most pervasive messenger RNA modification in the interior of eukaryotes (58). m^6^A RNA modification is dynamic and reversible under the control of m^6^A regulators and is divided into writers, erasers and readers by function. With rapid advances in high-throughput sequencing technology, studies on the function of m^6^A RNA modification in AML have made tremendous progress and triggered widespread interest, especially in LSCs maintenance and immune regulation.

### 3.1 The Regulation of m^6^A RNA Modification

#### 3.1.1 Writers (Methyltransferases)

Writers, namely, m^6^A methyltransferases, act as the transferrer of m^6^A to RNA adenosine in the form of a complex, including METTL3, METTL14, and WTAP. METTL3 interacts with METTL14 to constitute a heterodimer complex that is stable and difficult to interrupt. METTL3 has the catalytic function of adding m^6^A to RNA, while METTL14 is considered a scaffold to sustain the structure of METTL3 and to facilitate the binding of RNA. The METTL3-METTL14 complex coordinately induces RNA methylation (59). Despite being a WT-1-related protein, WTAP plays a role in m^6^A modification independent of WT1. In this process, WTAP is essential for localization of the METTL3-METTL14 complex in nuclear speckles and binding with target mRNAs (60). Despite its critical role in nuclear localization and recruitment of the METTL3-METTL14 complex, WTAP actually has no catalytic ability (61).

Furthermore, zinc finger CCCH-type containing 13 (ZC3H13), RNA binding motif protein 15/15B (RBM15/RBM15B) and vir-like m^6^A methyltransferase associated (VIRMA/KIAA1429) are also common writers. ZC3H13 functions as an anchor for WTAP-Virilizer-Hakai in nuclear speckles and regulates RNA m^6^A methylation, contributing to the self-renewal of mouse embryonic stem cells (62). RBM15 and RBM15B, with structural similarity and functional complementarity, possess peculiar domains that can bind the WTAP-METTL3 complex to target mRNA to induce m^6^A formation and can also bind the lncRNA X-inactive specific transcript (XIST) to facilitate XIST-mediated gene transcriptional silencing (63). VIRMA, also named KIAA1429, regulates preferential mRNA region-specific methylation in the 3′UTR and near the stop codon, by recruiting the catalytic complex METTL3/METTL14/WTAP (64).

Additionally, a series of new m^6^A writers have been discovered successively, for instance, methyltransferase-like 5 (METTL5), methyltransferase-like 16 (METTL16), NOP2/Sun RNA methyltransferase 2 (NSun2), phosphorylated CTD interacting factor 1 (PCIF1), zinc finger CCHC type containing 4 (ZCCHC4) and HAKAI. METTL5, as a heterodimer complex with TRMT112, cooperates with ZCCHC4 to deposit m^6^A on human 18S and 28S ribosome RNAs (rRNAs) (65). METTL16 participates in U6 small nuclear RNA (snRNA) methylation and contributes to controlling the homeostasis of S-adenosylmethionine (SAM), the methyl donor, by regulating the expression of MAT2A. Beyond that, METTL16 is also essential for common m^6^A RNA methylation modification (66). NSun2 is engaged in miRNA methylation to reduce the expression of miR-125b, resulting in cancer cell migration (67). PCIF1 exclusively acts as mammalian mRNA m^6^A_m_ methyltransferase, contributing to suppressing translation activity (68).The type E3 ubiquitin ligase HAKAI, also called CBLL1, is essential for stabilization of the m^6^A-METTL-associated complex (MACOM), playing a critical role in m^6^A deposition in Drosophila (69).

#### 3.1.2 Erasers (Demethylases)

Erasers, also called demethylases, are responsible for removing m^6^A methylation from the target mRNA. The coaction of methyltransferases and demethylases maintains m^6^A modification in a reversible and dynamic state. Two prominent erasers, FTO and ALKBH5, were successively discovered. FTO, belonging to the AlkB family of proteins, is the first to demethylate m^6^A in nuclear speckles of mRNA in an iron(II)- and α-KG-dependent manner, affecting the level of m^6^A (70). FTO predominantly targets nuclear m^6^A in a majority of cells and both cytoplasmic m^6^A and m^6^A_m_ in mRNA (71). FTO can also affect the transcriptional level by regulating m^6^A demethylation in mRNA and influence translation by mediating m^1^A demethylation in tRNA. Furthermore, FTO can regulate the demethylation of m^6^A in U6 RNA and m^6^A_m_ in snRNAs (71). As a first gene for non-syndromic human obesity, FTO is reported to participate in modulating adipogenesis *via* m^6^A demethylation (72).

ALKBH5, another demethylase belonging to the AlkB family, can exert catalytic effects to remove m^6^A from mRNA, regulating the abundance of m^6^A, mRNA export and RNA metabolism (73). Another member of the AlkB family, AlkB homolog 3 (ALKBH3), plays a central role in m^1^A and m^3^C demethylation in RNA and mediates m^6^A demethylation in tRNA to enhance protein translation (74).

#### 3.1.3 Readers (m^6^A Binding Proteins)

Readers, known as m^6^A binding proteins, are indispensable for m^6^A to take effect. YTH domain family proteins (YTHDF1, YTHDF2 and YTHDF3) and YTH domain-containing proteins (YTHDC1 and YTHDC2), both belonging to the YT521-B homology (YTH) domain family, are widely known as m^6^A readers that recognize and bind to the m^6^A methylation site, affecting RNA fate and gene expression (75, 76). Until now, YTHDC2 and all YTH domain family proteins have been found to be located in the cytoplasm, while the uniquely known m^6^A binding protein of the YTH domain family, which is located in the cell nucleus, is YTHDC1 (76–78). YTHDF1 directly enhances the translational efficiency in the manner of m^6^A methylation by interacting with initiation factors (eIFs) and promoting ribosomes uptake of its target mRNA in the cytoplasm (79). YTHDF2 can selectively recognize m^6^A to impact protein translation and RNA metabolism, with the C-terminus binding to m^6^A- methylated mRNA and the N-terminus conveying the YTHDF2-mRNA complex to RNA degradation sites (80). YTHDF3, another cytoplasmic m^6^A reader of YTH domain family proteins, fascinates translation by collaborating with YTHDF1 but not interacting with eIFs and promotes degradation of methylated mRNAs together with YTHDF2, both in an m^6^A-dependent manner (81). YTHDC1, as the only reader in the nucleus, plays a part in transcription and gene expression by selectively binding to methylated RNA (82). YTHDC1 plays a vital role in directly regulating mRNA splicing in the form of accelerating exon inclusion in targeted mRNAs through enhancing SRSF3 but repressing SRSF10 RNA-binding ability (83). YTHDC1 is also engaged in nuclear export by promoting RNA binding to both SRSF3 and NXF1 in an m^6^A-dependent manner but independent of pre-mRNA splicing (84). YTHDC2, the only RNA helicase-containing m^6^A binding protein, can improve translation efficiency through m^6^A methylation in mRNA coding regions (CDS) and interaction with the small ribosomal subunit (85, 86). Furthermore, YTHDC2 can regulate the degradation of target mRNAs by collecting RNA decay factors (86).

**Figure 1:**
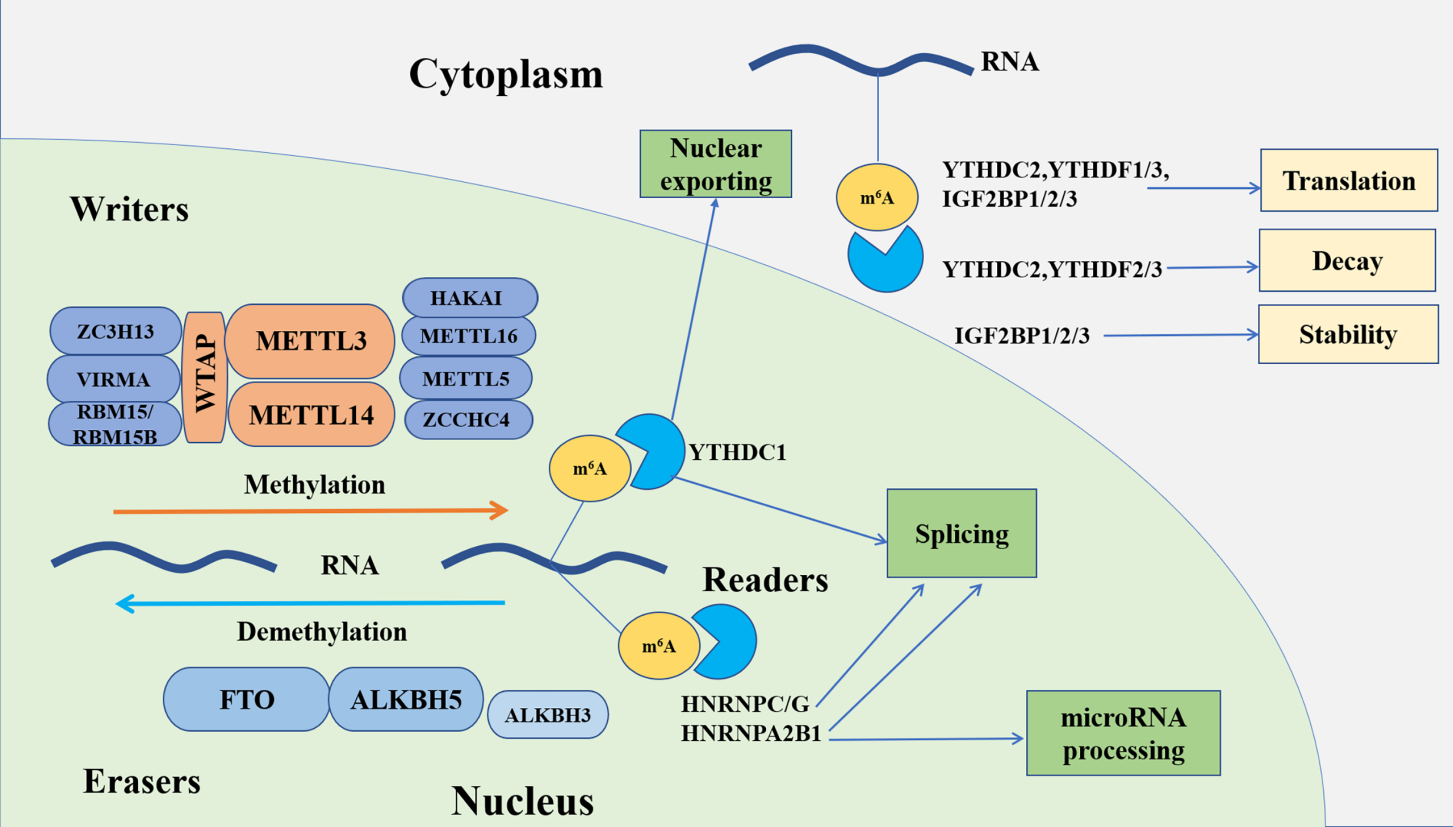
The functions of m^6^A RNA modification. m^6^A writers and erasers reversibly and dynamically regulate the m^6^A methylation modification. Readers can recognize and bind to the m^6^A methylation site to influence RNA fate, including nuclear exporting, splicing, stability, decay, and translation, as well as microRNA processing.

In addition to the aforesaid m^6^A readers, which belong to the YTH domain family, there are two new families recognized as m^6^A binding proteins, the insulin-like growth factor 2 mRNA-binding protein (IGF2BP) family and the heterogeneous nuclear ribonucleoprotein (HNRNP) family. IGF2BPs, including IGF2BP1, IGF2BP2 and IGF2BP3, play a central role in enhancing the stability of mRNA and protein translation by recognizing m^6^A with K homology (KH) domains and can even interact with MYC to accelerate tumorigenesis in an m^6^A-dependent manner (87). HNRNPA2B1, one member of the HNRNP family, functions as a nuclear m^6^A ‘reader’ to directly bind the m^6^A mark, contributing to promoting pri-miRNA transcription by binding the microRNA microprocessor complex protein DGCR8 and regulating alternative splicing (88). HNRNPC and HNRNPG, two other members of the HNRNP family, both interact with target mRNAs by remodeling the RNA structure in an m^6^A-dependent way, changing the abundance and alternative splicing of target mRNAs (89, 90). The major roles of m^6^A regulators are summarized in **Figure 1**.

### 3.2 The m^6^A Modification in AML and LSCs

Currently, a variety of studies on the roles of m^6^A modification in leukemia have been conducted, displaying that m^6^A modification not only participates in the proliferation, colony formation, differentiation and apoptosis of leukemia cells but also maintains the pluripotency and self-renewal of LSCs.

METTL3 is indispensable for the development of AML cells due to the CAATT box binding protein CEBPZ in the transcriptional start site (TSS) binding chromatin, independent of METTL14. Promoter-bound METTL3 facilitates mRNA transcription and protein translation to maintain the growth of AML cells in an m^6^A-dependent manner. METTL3-knockdown cells exhibit distinct cell cycle arrest and marked differentiation, especially MLL-AF9-driven AML cells (91). METTL3 mRNA is highly expressed in human AML samples compared to other types of tumors. Increased METTL3 in leukemic cells is required for the maintenance of differentiation arrest of AML cells by enhancing transcripts of c-MYC, BCL-2 and PTEN and inactivating the PI3K/AKT pathway, accompanied by elevated m^6^A abundance. Loss of METTL3 in AML cells can induce apoptosis and enhance differentiation, impeding leukemic cell development (23). METTL3 also participates in regulating WTAP mRNA translation and protein stabilization to maintain WTAP protein homeostasis, which is recognized as an oncogenic protein in AML (92, 93). STM2457, a small-molecule METTL3 inhibition, can suppress the development and promote the differentiation and apoptosis of AML cells *in vivo* and *in vitro*. After treatment with STM2457, the CD93+ cell population, which is identified as leukemia stem cells, was significantly reduced, while the CD48+ intensity was apparently increased (94, 95). METTL3 inhibition annihilates LSCs, interrupting AML growth and propagation. We turn our attention to METTL3 as an active participant in the self-renewal of LSCs.

METTL14 plays a pivotal role in repressing AML differentiation and maintaining the self-renewal of LSCs by modulating the mRNA stability and translation of MYC and MYB in an m^6^A-dependent manner, while METTL14 expression is suppressed by SPI1, a master transcription factor (TF). In other words, METTL14 exerts an effect on AML initiation and development *via* the SPI1-METTL14-MYC/MYB signaling axis (24). METTL14 can also interact with chimeric mRNA to inhibit myeloid cell differentiation through complexes composed of fusion protein and MALAT1, a nuclear speckle-specific long noncoding RNA, depending on YTHDC1 and SRSF3 (96).

WTAP is engaged in promoting AML proliferation and differentiation arrest through the mammalian target of rapamycin mTOR signaling pathway, including its downstream effector p70 ribosomal subunit 6 kinase (pS6K), which functions as an oncogenic protein (93). WTAP is considered a poor prognostic factor by enhancing leukemogenesis, the cell cycle, and chemoresistance of AML cells based on m^6^A modification of MYC mRNA (97). Whether WTAP influences the self-renewal of LSCs remains elusive.

FTO, as an oncogenic protein, participates in promoting AML cell proliferation and transformation while hindering AML cell differentiation and apoptosis, especially in AML subtypes with MLL-rearrangements and PML-RARA, FLT3-ITD and NPM1 mutations. ASB2 and RARA, which play a part in hematopoietic cell differentiation, act as negative target genes of FTO in AML. FTO plays a carcinogenic role by inversely regulating ASB2 and RARA both at the RNA and protein levels in an m^6^A-dependent way (22). R-2-hydroxyglutarate (R-2HG), an antimetabolite generated by mutant isocitrate dehydrogenase 1/2 (IDH1/2), can target FTO to exert an anti-leukemia effect. By negatively regulating FTO, R-2HG inhibits MYC/CEBPA-related pathways and impedes lactate dehydrogenase B (LDHB) and phosphofructokinase platelet (PFKP) to abrogate aerobic glycolysis, both through a mechanism dependent on m^6^A (98, 99). In addition, FTO mediates the resistance to tyrosine kinase inhibitors (TKIs) in leukemia cells by regulating MERTK and BCL-2 through m^6^A demethylation (100). Upon FTO knockdown or treatment with the FTO inhibitors CS1 or CS2, the CD34+ LSC proportion was significantly decreased, indicating that FTO facilitates the self-renewal of LSCs by targeting MYC and CEBPA (101). The dramatically reduced number of CD34+CD38- LSCs in a PDX AML mouse model after treatment with the FTO inhibitor FB23-2 is another example demonstrating that FTO is associated with the self-renewal of LSCs (102).

ALKBH5 is reported to play key roles in the initiation of AML cells and self-renewal of LSCs in an m^6^A-dependent manner, predicting poor prognosis in AML patients. ALKBH5 directly regulates the mRNA stability of its target gene TACC3, which affects MYC and p21, contributing to leukemogenesis and the self-renewal of LSCs (25). ALKBH5 is affected by chromatin alterations attributed to decreased H3K9me3 and positively regulated by MYB and polymerase II (Pol II) in AML cells. KDM4C is the root cause of all these regulators influencing ALKBH5 expression, resulting in the progression of AML cells and the self-renewal of LSCs. ALKBH5 fine-tunes the stability of AXL mRNA depending on m^6^A modification mediated by YTHDF2, promoting the development and clonogenic potential of leukemia cells (103). Collectively, ALKBH5 plays essential roles in LSC self-renewal and maintenance through a variety of mechanisms.

YTHDF2, overexpressed in human AML samples, is considered to facilitate leukemia initiation and LSC maintenance by shortening the half-life of various m^6^A transcripts in favor of LSC functions and regulating mRNA decay in an m^6^A-dependent way. YTHDF2 depletion makes AML cells more susceptible to TNF, inducing increased apoptosis (104). The AML1/ETO-HIF1α loop participates in regulating YTHDF2 to promote leukemia cell development in t (8;21) AML cells. YTHDF2 downregulation can increase m^6^A abundance and TNF receptor superfamily member 1b (TNFRSF1b) expression, contributing to apoptosis (105). YTHDC1, a nuclear reader, plays a part in facilitating the initiation and maintenance of AML cells, especially blocking differentiation, by binding to m^6^A to form dynamic nuclear YTHDC1-m^6^A condensates (nYACs) mediated by liquid-liquid phase separation (LLPS). nYACs play a crucial role in maintaining the stability of the target mRNA MYC, which is directly regulated by YTHDC1 in an m^6^A-dependent pathway, by preventing RNA degradation by the polyA tail exosome targeting complex (PAXT) (106). In addition to proliferation and colony formation, YTHDC1 also accounts for LSC self-renewal by enhancing the stability of minichromosome maintenance 4 (MCM4), which contributes to DNA replication (107). IGF2BP1 expressed in AML cells promotes leukemogenesis and proliferation and maintains the stemness of leukemia cells by regulating HOXB4, MYB, and ALDH1A1, which are LSC phenotype-associated transcriptional and metabolic factors. IGF2BP1 depletion can reduce colony formation, impede leukemogenesis and proliferation, and postpone leukemia cell development in NSG mice. IGF2BP1 functions as an oncogenic protein and regulator of LSC self-renewal maintenance in an m^6^A-dependent manner (108). IGF2BPs interact with YBX1, an RNA-binding protein (RBP), to facilitate YBX1 stabilization of MYC and BCL-2 mRNA in an m^6^A-depentdent way to maintain AML cell development (109). Based on this finding, IGF2BPs indirectly take part in the initiation and maintenance of AML cells.

Taken together, m^6^A modification, mediated by writers, erasers or readers, is indispensable for AML cell initiation, proliferation, growth, differentiation and survival. Furthermore, m^6^A regulators play a critical role in LSC self-renewal, which is responsible for AML recurrence and chemoresistance.

### 3.3 The m^6^A Modification in the TIME

The roles of m^6^A modification in the TIME have increasingly attracted extensive attention. METTL3 or METTL14 depletion promotes immune responses to anti-PD-1 therapy in colorectal cancer (CRC) by increasing CD8+ T cell infiltration, cytokine secretion and chemokine production in the TME. METTL3 and METTL14 expressed on tumor cells remodel the TIME by regulating immune cells, cytokines and chemokines through the IFN-c-Stat1-Irf1 pathway in an m^6^A-dependent manner (110). FTO enhances the expression of PD-L1 in colon cancer cells through m^6^A methylation modification but not the IFN-g signaling-dependent pathway (111). In addition, FTO represses T cell immune activation in the lung cancer cell lines and melanoma cells by regulating JunB and C/EBPb in an m^6^A-dependent way to accelerate tumor glycolysis. The FTO inhibitor Dac51 improves T cell infiltration and cytotoxic capacity cooperatively with an anti-PD-L1 antibody (112). ALKBH5 in tumor cells recruits immune suppressive cells, including myeloid-derived suppressor cells (MDSCs) and Tregs, and facilitates lactate accumulation to regulate the anti-PD-1 therapy response depending on m^6^A in melanoma. ALKBH5 inhibitor promotes anti-tumor immunity of anti-PD-1 (113). ALKBH5, induced by hypoxia in glioblastoma multiforme (GBM), is responsible for tumor-associated macrophage (TAM) recruitment, leading to a suppressive TIME by facilitating CCX8/IL-8 secretion (114).

At present, studies on m^6^A modification in the TIME of AML have been successively performed. FTO is reported to induce the expression of immune checkpoint leukocyte immunoglobulin-like receptor subfamily B 4 (LILRB4), which contributes to T cell suppression and tumor infiltration in AML, leading to immune evasion (115).Upon treatment with decitabine, FTO expression was significantly increased, resulting in decreased m^6^A abundance in AML cells. Meanwhile, the immune checkpoints LILRB4, PD-L1, PD-L2 and PD-1 in AML cells or T cells were also elevated, especially LILRB4, which was well above the others. With FTO knockdown or FTO inhibitors, LIRLB4 expression was dramatically reduced at both the RNA and protein levels through YTHDF2-mediated m^6^A modification (101). This may be a mechanism by which HMAs induce immune evasion and chemoresistance. After pretreatment with the FTO inhibitors CS1 or CS2, AML cells with high expression of LILRB4 were subsequently co-cultured with activated T cells, displaying that LILRB4 expression was significantly decreased and that the killing effect of T cells on AML cells was remarkably enhanced (101). Conceivably, FTO-associated m^6^A modification plays pivotal roles in immunoregulation in AML.

The gold nanorods GNRa-CSP12 regulates FTO and ALKBH5, which are both Fe2+-dependent demethylases, to impede the proliferation of AML cells by triggering ferroptosis in an m^6^A-dependent manner. Upon GNRa-CSP12 treatment, genes of immune checkpoint pathways are downregulated, which is ascribed to the decreased stability of SLC2A3, CD276, and PKM transcripts. GNRa-CSP12 can also overcome FTO-mediated TKI resistance. In addition, GNRa-CSP12 plays an important role in promoting the antileukemia efficacy of PD-L1 antibody by targeting FTO and ALKBH5 to activate T cell responses, such as the infiltration of CD4+ T cells and CD8+ T cells, as well as the cytotoxic cytokine IFN-γ in the TIME (116). This shows that m^6^A modification mediated by FTO and ALKBH5 participates in the regulation of the immune response in AML.

Given that genes involved in immune response processes are downregulated after YTHDF2 depletion in AML cells, YTHDF2 is perceived to mediate the immune response (104). The detailed mechanism by which YTHDF2 regulates the immune response in AML remains to be elucidated.

Du et al. divided m^6^A modification patterns into three distinct clusters based on 23 m^6^A regulators in 255 AML specimens derived from public databases, and calculated m^6^A scores with statistical software, indicating that different clusters were corresponded with different immune infiltration phenotypes in the TIME. They found that the expression of immune checkpoints, including PD-L1, PD-L2, MRP1, and MRP2, was remarkably higher in AML patients with lower m^6^A scores, implying that m^6^A modification takes part in antileukemia immunity. Additionally, m^6^A scores are positively correlated with immune response and better prognosis (117). In brief, m^6^A modification plays significant roles in regulating the TIME and affecting immunotherapy efficacy. m^6^A-related long noncoding RNAs (lncRNAs) are reported to influence the immune response in AML (118). It can be assumed that m^6^A indirectly modulates the immune response by interacting with lncRNAs.

**Figure 2:**
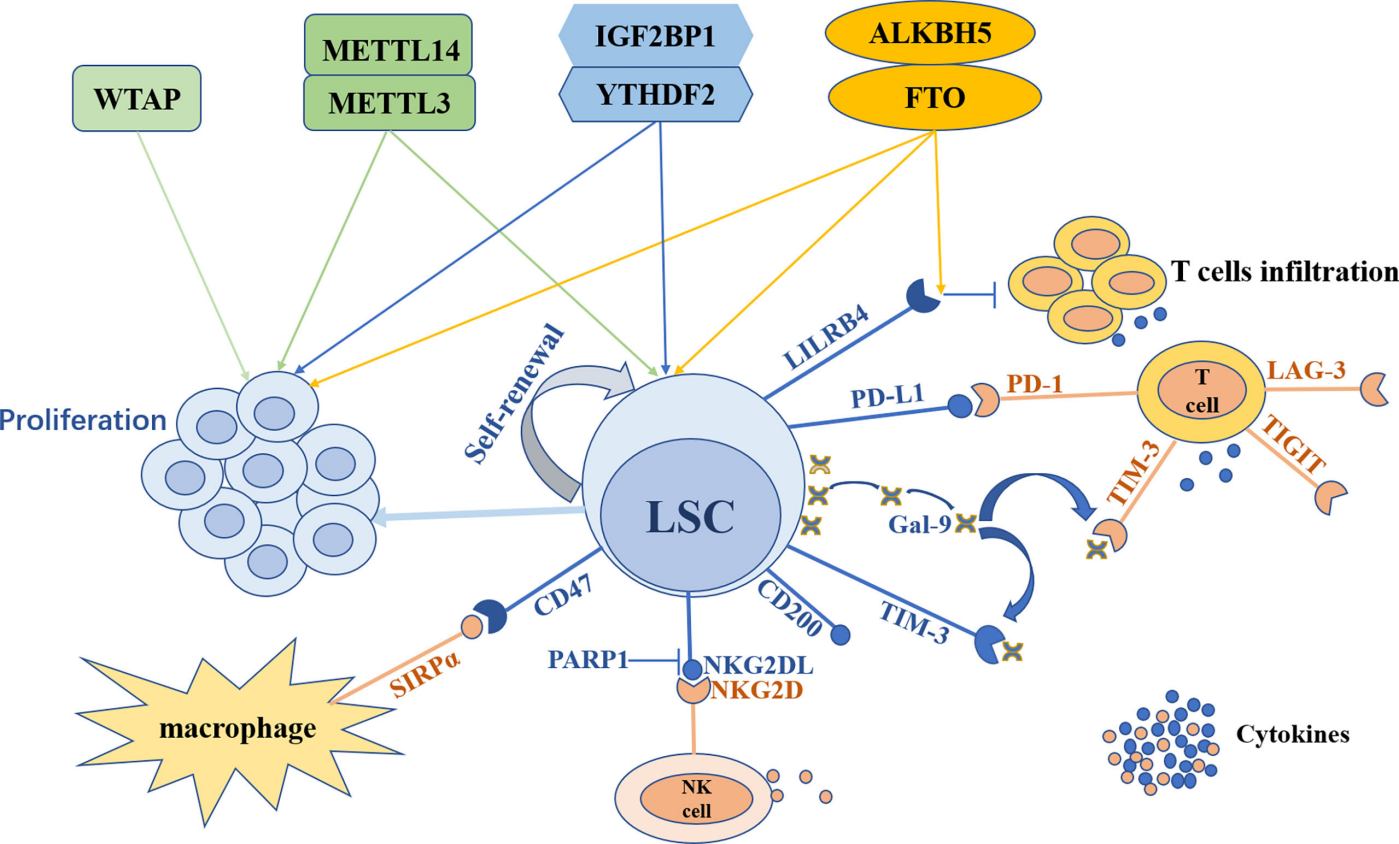
The outline of m^6^A modification in leukemia stem cells and the tumor immune microenvironment. Leukemia stem cells (LSCs) can maintain self-renewal and trigger leukemia cells proliferation. LSCs can escape from the surveillance and elimination of the immune system through various mechanisms. With the expression of TIM-3, LSCs secrete galectin-9 (Gal-9) to form TIM-3/Gal-9 autocrine loop. Meanwhile Gal-9 combines with TIM-3 expressed on T cells to inhibit T cells immunity. Furthermore, LSCs express PD-L1 to recognize and bind its receptor PD-1 on T cells to impede the killing of T cells. LSCs can also express LILRB4 and CD200 to suppress T cells activity. In addition, LSCs can express CD47 to combine with SIRPα on macrophages to escape from innate immune attacks and can suppress NKG2DL expression through PARP1 to avoid NK cells killing. m^6^A modification both participates in the regulation of LSCs and the TIME. METTL3, METTL14, FTO, ALKBH5, YTHDF2 and IGF2BP1 can both promote LSCs self-renewal and leukemia cells proliferation, while WTAP can only enhance leukemia cells proliferation. FTO regulates the expression of LILRB4 on LSCs to suppress T cells infiltration and cytokines secretion in an m^6^A-dependent way.

Compared to solid tumors, studies on m^6^A modification in the TIME of AML are limited. However, it sheds further insight into future research to explore the impact of m^6^A on the immune microenvironment and to demonstrate why the therapeutic effect of immunotherapy alone is unsatisfactory in AML, paving the way to develop novel treatment strategies for AML patients. The outline of m^6^A modification in LSCs and the TIME is shown in **Figure 2**.

## 4 Clinical Application of m^6^A Modification

m^6^A regulators, such as METTL3, METTL14, WTAP, FTO, ALKBH5, and YTHDF2, are highly expressed in a variety of AML subtypes. The expression of METTL3 mRNAs is apparently increased in M0, while METTL14 is increased in M1 and M3 (92). MTTL14 is found to be much more highly expressed in AML with t (11q23), t (8;21), or t (15;17) (24). WTAP is significantly overexpressed in AML with FLT3-ITD and/or NPM1 mutations but not with t (15;17) and can induce chemoresistance during AML treatment (93, 97). Accompanied by complete remission (CR), WTAP expression is decreased (97). FTO is remarkably overexpressed in AML with t (15;17) and t (11q23), as well as FLT3-ITD and/or NPM1 mutations. Moreover, FTO is responsible for TKI resistance in leukemia cells, suggesting that targeting FTO may reverse TKI resistance (100). In AML carrying t (8,21), inv (16), and t (11q23), as well as normal karyotypes, ALKBH5 expression is relatively high (103). Genetic alterations of encoding genes, which are associated with the m^6^A writer complex, are negatively associated with prognosis in AML patients (23). WTAP is a poor prognostic risk factor in AML patients, resulting in a shorter overall survival (97). Similarly, ALKBH5 can also predict poor prognosis, and higher ALKBH5 expression is consistent with a high rate of relapse and chemoresistance (103). IGF2BP1 triggers chemoresistance through LSC phenotype-related regulators, including HOXB4, MYB, and ALDH1A1, and is correlated with prognosis (108). Based on these studies, m^6^A writers, erasers, and readers show promise as diagnostic and prognostic markers in AML patients.

m^6^A methylation plays vital roles in LSCs and the TIME, which can interplay with each other, leading to relapse and chemoresistance in AML. Many studies have confirmed that m^6^A writers, erasers, and readers are instrumental for myeloid leukemogenesis and maintaining LSC self-renewal. Meanwhile, FTO, ALKBH5 and YTHDF2 are reported to regulate the antileukemia immune response by altering the TIME (101, 104, 116). Hence, m^6^A regulators are expected to be potential therapeutic targets in AML. These studies shed new light on the treatment of AML by targeting m^6^A modification to eradicate LSCs and to increase the efficiency of immunotherapy.

At present, there have been several studies on targeting m^6^A for AML treatment. Small-molecule inhibitors of METTL3, STM2457 and UZH1a, can prohibit the proliferation of varying AML cell lines. STM2457 can also decrease the number of LSCs marked CD93+, weaken clonogenic capacity and accelerate apoptosis. Furthermore, STM2457 restrains the self-renewal of LSCs to hinder AML cell implantation and propagation in human PDX models (94, 95). This sheds further insight into a potential therapeutic window in which depletion of METTL14 expression can repress the self-renewal of LSCs while exerting a relatively weak effect in normal hematopoietic stem and progenitor cells (HSPCs). Upon treatment with all-trans retinoic acid (ATRA) or PMA, which are differentiation-inducing agents, the levels of METTL14 and m^6^A were also reduced (24). It is promising that the therapeutic regimen combing a METTL14 inhibitor with ATRA or ATO may have a synergistic effect.

Owing to the inverse effect on the differentiation of FTO, depletion of FTO consequently triggers ATRA-induced cell differentiation, significantly increasing the differentiated population of NB4 cells (22). Based on this conclusion, we can speculate that the therapeutic strategy combining with FTO inhibition with ATRA may improve the treatment effect of acute promyelocyte leukemia (APL). R-2HG plays an important role in anti-leukemia activity by targeting FTO through various mechanisms (98, 99). It provides a potential therapeutic window for combinational application with R-2HG, used as a nonspecific FTO inhibitor, and other agents, such as IDH inhibitor or HMAs or standard chemotherapy. To date, there are various FTO inhibitors with anti-leukemia activity in AML, including the natural compounds radicicol, Saikosaponin D, FB23-2, CS1 and CS2 (101, 102, 119, 120).Among them, FB23-2, CS1, and CS2 can reduce the numbers of LSCs. Furthermore, CS1 and CS2 can also surmount immune evasion induced by HMAs and make AML cells more susceptible to activated T cells (101). This striking finding paves the way to improve the therapeutic effect of AML or myelodysplastic syndromes (MDS) in combination with FTO inhibitors or anti-LILRB4 monoantibody based on HMAs.

ALKBH5 is engaged in the self-renewal and maintenance of LSCs, without influencing normal hematopoietic stem cells (HSCs), implying that ALKBH5 is expected to be a promising therapeutic target for LSC eradication (25, 103). To date, two low micromolar active ALKBH5 inhibitors have exhibited certain effects on the AML cell line HL-60 (121). Similar to ALKBH5, targeting YTHDF2 does not harm normal hematopoiesis, which makes YTHDF2 a feasible target to eliminate LSCs (104).

It’s worth noting that whether there are potential side effects to target m^6^A modulators in view of their important roles in normal biological process. The security of METTL3 inhibitor STM2457 has been demonstrated *in vivo*, with no apparent impact on body weight and normal hematopoiesis (95). Su and his colleagues have assessed the potential drug toxicity of FTO inhibitors, CS1 and CS2, in C57BL/6 mice. They found that the differences of complete blood count, Haemotoxylin and Eosin staining, whole body or organ weight between the drug-treated groups and control group are not significant (101).The toxicity of FTO inhibitor FB23-2 has been also evaluated in BALB/c mice. There are no obvious toxic side effects in FB23-2-treated group compared to control group, such as body weight loss, organ lesion and hematopoietic damage (102).The preclinical results showed that the potential side effects of therapy targeted m^6^A modulators is mild. But the safety of clinical application needs to be confirmed in more clinical trials.

Taking a broad view, targeting m^6^A opens a new door to treat AML by eliminating LSCs and regulating the immune microenvironment. Additionally, targeting m^6^A therapy is used in combination with other drugs, such as HMAs, ATRA, ATO or immune checkpoint inhibitors, to improve the therapeutic efficacy in AML patients. The roles of m^6^A modification in leukemia cells in AML are summarized in **Table 1**, and the roles of m^6^A modification in the immune microenvironment of AML are summarized in **Table 2**.

**Table 1:** The roles of m^6^A modification in leukemia cells in acute myeloid leukemia.

m^6^A regulators	Roles in leukemia cells	Target genes	Upstream	Inhibitors	Refs.
METTL3	Promoting growth and proliferation, as well as inhibiting differentiation	SP1	CEBPZ	No study	(91)
METTL3	Enhancing colony formation and proliferation, as well as impeding differentiation and apoptosis	c-MYC, BCL-2, PTEN	No study	No study	(23)
METTL3	Promoting growth, inhibiting differentiation and apoptosis and maintaining LSCs	No study	No study	STM2457	(95)
METTL14	Promoting proliferation, inhibiting differentiation and apoptosis, as well as maintaining self-renewal of LSCs	MYB MYC	SPI1	No study	(24)
WTAP	Promoting proliferation, inducing chemoresistance and inhibiting differentiation	No study	No study	No study	(93)
WTAP	Regulating leukemogenesis, proliferation, cell cycle, differentiation and chemoresistance	No study	No study	No study	(97)
FTO	Enhancing leukemogenesis and proliferation, as well as repressing differentiation and apoptosis	ASB2 RARA	No study	No study	(22)
FTO	Promoting proliferation, regulating cell cycle, and inhibiting apoptosis	MYC CEBPA	No study	R-2HG	(98)
FTO	Promoting leukemogenesis and proliferation through aerobic glycolysis	PFKP LDHB	No study	R-2HG	(99)
FTO	Promoting proliferation, inhibiting apoptosis and inducing TKIs resistance	MERTK BCL-2	No study	No study	(100)
FTO	Promoting proliferation, as well as inhibiting apoptosis and differentiation	ASB2 RARA MYC CEBPA	No study	FB23-2	(102)
FTO	Promoting self-renewal of LSCs	LILRB4	No study	CS1 CS2	(101)
ALKBH5	Maintaining self-renewal of LSCs and promoting proliferation, as well as inhibiting apoptosis	TACC3	No study	No study	(25)
ALKBH5	Promoting leukemogenesis and maintainning LSCs	AXL	KDM4C MYB Pol II	No study	(103)
YTHDF2	Promoting proliferation and self-renewal of LSCs	TNFR2	No study	No study	(104)
YTHDF2	Promoting leukemogenesis and maintainning LSCs	TNFRSF1b	AML1/ETO-HIF1α axis	No study	(105)
YTHDC1	Promoting leukemogenesis and impeding differentiation	MYC	No study	No study	(106)
IGF2BP1	Promoting leukemogenesis and proliferation,inhibiting differentiation, inducing chemoresistance and maintainning LSCs properties	HOXB4 MYB ALDH1A1	No study	No study	(108)

**Table 2:** The roles of m^6^A modification in the immune microenvironment of acute myeloid leukemia.

m^6^A regulators	Roles in the immune microenvironment	Target genes	Refs.
FTO	Upregulating immune checkpoint LILRB4 to induce immune evasion and decitabine resistance; further to support tumor cell infiltration into tissues and suppress T cell activity *via* a signaling pathway that involves APOE, LILRB4, SHP-2, uPAR and ARG1 in acute myeloid leukemia cells	LILRB4	(101) (115)
FTO	Maintaining the stability of SLC2A3, CD276, and PKM transcripts, to upregulating genes of immune checkpoint pathways	SLC2A3 CD276 PKM	(116)
ALKBH5	Maintaining the stability of SLC2A3, CD276, and PKM transcripts, to upregulating genes of immune checkpoint pathways	SLC2A3 CD276 PKM	(116)
YTHDF2	Maintaining the stability of m^6^A-modified transcripts, to decrease the half-life of LILRB4 mRNA	LILRB4	(101)
YTHDF2	Upregulation genes involved in immune response processes	No study	(104)

## 5 Discussion

In summary, LSCs and the TIME cooperatively promote AML initiation and progression, contributing to relapse and chemoresistance in patients with AML through diverse mechanisms. m^6^A methylation, as a burgeoning RNA epigenetic modification, plays significant roles in leukemogenesis, proliferation, differentiation, apoptosis, and LSC self-renewal by regulating target mRNA stability and protein translation efficiency. To date, m^6^A writers (METTL3, METTL14, WTAP), erasers (FTO, ALKBH5), and readers (YTHDF2, YTHDC1, IGF2BP1) are considered oncogenic proteins in AML and are responsible for the maintenance of LSCs. METTL14, WTAP, and FTO are highly expressed in specific AML subtypes. Moreover, WTAP, ALKBH5, and IGF2BP1 are correlated with prognosis and chemoresistance. Based on the functions of m^6^A in AML, there have been some inhibitors targeting m^6^A, such as the METTL3 inhibitors STM2457 and UZH1a and the FTO inhibitors FB23-2, CS1 and CS2. With no or relatively less impact on HSCs, targeting METTL14, ALKBH5, or YTHDF2 is promising for eradicating LSCs and reducing relapse and chemoresistance in AML patients. In addition, combination inhibitors targeting m^6^A with other therapeutic agents, such as HMAs, ATRA, ATO, immune checkpoint blockade, and standard chemotherapy, are anticipated to be innovative therapeutic regimens. Therefore, m^6^A regulators can be regarded as novel biomarkers for AML diagnosis, prognosis, and response evaluation, as well as attractive targets to improve the therapeutic effect in AML patients. However, large-scale clinical studies are needed to demonstrate the efficacy and safety of targeting m^6^A in AML patients.

In addition, m^6^A modification also takes part in remodeling the TIME by influencing immune cell infiltration, cytokine secretion, and immune checkpoint expression. Among m^6^A regulators, FTO is well studied in immune regulation, mediating HMAs resistance and immune evasion in AML. To date, studies on the functions of m^6^A modification in the AML immune microenvironment, especially in the crosstalk between LSCs and the TIME, are just the tip of the iceberg. Therefore, more related studies are required to explore the roles and mechanisms.

Taken together, m^6^A modification not only maintains the stemness of leukemia cells but also regulate the immune microenvironment in AML. In view of this feature, targeting m^6^A is promising to kill two birds with one stone. It also provides a novel opportunity to refine diagnosis, prognosis stratification and therapeutic efficacy evaluation for AML patients. It sheds new light on precision medicine for AML patients.

## Author Contributions

XO and YG contributed to the design of the review. The manuscript was written by XO. All authors contributed to the article and approved the submitted version.

## Funding

The work was supported by the Foundation of the Science and Technology Department of Sichuan Province (NO.2019YFS0026).

## Conflict of Interest

The authors declare that the research was conducted in the absence of any commercial or financial relationships that could be construed as a potential conflict of interest.

## Publisher’s Note

All claims expressed in this article are solely those of the authors and do not necessarily represent those of their affiliated organizations, or those of the publisher, the editors and the reviewers. Any product that may be evaluated in this article, or claim that may be made by its manufacturer, is not guaranteed or endorsed by the publisher.

## Glossary

**Table:**

AML	acute myeloid leukemia
LSCs	leukemia stem cells
TIME	tumor immune microenvironment
m^6^A	N6 methyladenosine
HMAs	hypomethylating agents
HSCT	hematopoietic stem cell transplantation
CSCs	cancer stem cells
FTO	fat mass and obesity associated protein
METTL3	methyltransferase-like 3
METTL14	methyltransferase-like 14
WTAP	Wilm’s tumor 1-associated protein
ALKBH5	AlkB homolog 5
TIM-3	T cell immunoglobulin and mucin protein 3
Gal-9	Galectin-9
sTIM-3	soluble TIM-3
HIF1&alpha	hypoxia-inducible factor 1-alpha
PD-L1	programmed cell death-ligand 1
PARP1	Poly-ADP-ribose polymerase 1
NKG2DL	NKG2D ligand
TME	tumor microenvironment
ZC3H13	zinc finger CCCH-type containing 13
RBM15/RBM15B	RNA binding motif protein 15/15B
VIRMA/KIAA1429	vir-like m6A methyltransferase associated
XIST	lncRNA X-inactive specific transcript
METTL5	methyltransferase-like 5
METTL16	methyltransferase-like 16
NSun2	NOP2/Sun RNA methyltransferase 2
PCIF1	phosphorylated CTD interacting factor 1
ZCCHC4	zinc finger CCHC type containing 4
rRNAs	ribosome RNAs
snRNA	small nuclear RNA
SAM	S-adenosylmethionine
MACOM	m6A-METTL-associated complex
ALKBH3	AlkB homolog 3
YTH	YT521-B homology
eIFs	initiation factors
CDS	coding regions
IGF2BP	insulin-like growth factor 2 mRNA-binding protein
HNRNP	heterogeneous nuclear ribonucleoprotein
KH	K homology
TSS	transcriptional start site
TF	transcription factor
pS6K	p70 ribosomal subunit 6 kinase
R-2HG	R-2-hydroxyglutarate
IDH1/2	isocitrate dehydrogenase 1/2
LDHB	lactate dehydrogenase B
PFKP	phosphofructokinase platelet
TKIs	tyrosine kinase inhibitors
Pol II	polymerase II
TNFRSF1b	TNF receptor superfamily member 1b
nYACs	nuclear YTHDC1-m6A condensates
LLPS	liquid-liquid phase separation
PAXT	polyA tail exosome targeting complex
MCM4	minichromosome maintenance 4
CRC	colorectal cancer
MDSCs	myeloid-derived suppressor cells
GBM	glioblastoma multiforme
TAM	tumor-associated macrophage
LILRB4	leukocyte immunoglobulin-like receptor subfamily B 4
lncRNAs	long noncoding RNAs
HSPCs	hematopoietic stem and progenitor cells
ATRA	all-trans retinoic acid
APL	acute promyelocyte leukemia
MDS	myelodysplastic syndromes
HSCs	hematopoietic stem cells
